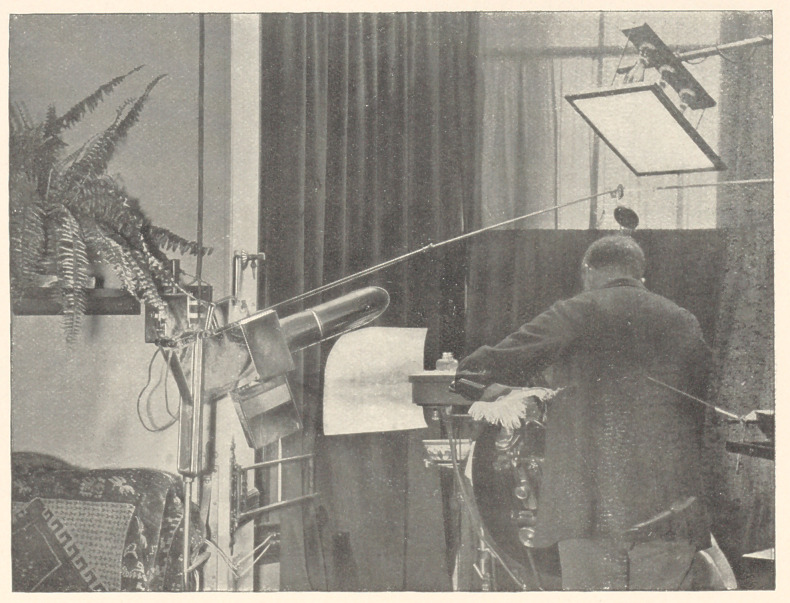# An Interesting Case

**Published:** 1896-06

**Authors:** S. L. Goldsmith

**Affiliations:** New York


### AN INTERESTING CASE.

BY DR. S. L. GOLDSMITH, NEW YORK.

Miss A., who at the time was undergoing some painful dental treatment, said, "Oh, if hypnotism were only advanced to such a stage that one could have the teeth filled without pain!" I replied that it was not an impossibility even now; in fact, that it had been done. The patient then expressed a desire for me to try it upon her, and with the precaution of having a third party present, I endeavored without success to hypnotize her. In the mean time I ascertained that the patient was a sufferer from insomnia, and, as I saw a greater object in view, I tried again at her next appointment and succeeded. The hypnosis was complete.

I excavated the cavities and, in fact, separated teeth with almost no pain. The method used was the so-called "mixed method," —that is, a combination of the suggestions of Bernheim and the strokings, etc., of Charcot.

Now, having found that the patient was a good subject, and as insomnia was out of my sphere of practice, I took her to a medical colaborer of mine, who at once examined her, and finding no organic disorder which could cause her trouble, ventured a very favorable prognosis with the treatment mentioned. The physician then put her into the hypnotic state and suggested to her that she would sleep the ensuing night from ten until 6.30 next morning. At 9.30 that evening the patient was so sleepy that she could no longer hold her book, and retired. Upon awakening she looked at her watch, the hands of which pointed to 6.35. For years her limit of sleep had been three hours a night. The physician has gradually lengthened the time over which the suggestion was to have effect, until now one suggestion has given her a proper night's rest every night for six weeks. I have no doubt that this can soon be lengthened to six months, and, in fact, that in a couple of years the suggestion will not be necessary at all.

Before the commencement of our treatment, Miss A. was a nervous, morose individual, who jumped every time the door-bell rang. Since the treatment she has materially gained in weight and is as happy as a well-nourished young lady should be.

## Figures and Tables

**Figure f1:**